# Nondestructive analysis of alterations of Chinese jade artifacts from Jinsha, Sichuan Province, China

**DOI:** 10.1038/s41598-020-73290-y

**Published:** 2020-10-28

**Authors:** Yi Bao, Xuemei Yun, Chaohong Zhao, Fang Wang, Yuesheng Li

**Affiliations:** 1 Institute of Archaeological Science, Fudan University, Shanghai, 200433 People’s Republic of China; 2 Department of Cultural Heritage and Museology, Fudan University, Shanghai, 200433 People’s Republic of China; 3 Research Center for the Development of Geosciences, China University of Geosciences (Beijing), Beijing, 100083 People’s Republic of China; 4 School of Archaeology and Museology, Peking University, Beijing, 100871 People’s Republic of China; 5 Chengdu Jinsha Site Museum, Chengdu, 610091 People’s Republic of China; 6 Department of Materials Science, Fudan University, Shanghai, 200433 People’s Republic of China

**Keywords:** Materials science, Optical spectroscopy

## Abstract

Jade, which is one of the most characteristic materials constituting Chinese artifacts, signifies cultural differences between ancient Chinese and western civilizations. One of the most important typical characteristics of ancient jade artifacts recovered through archeological excavations is color alterations due to human activity and natural weathering, which has led to an area of intensive research in archeology. “Alteration” refers to chemical component and structural changes in jade artifacts caused by human activity and natural weathering, which is different from the term in geology. In this study, Raman spectroscopy was used to analyze six color alterations on ancient jade artifacts unearthed from the Jinsha Site in Sichuan Province, a region famous for artifacts with colorful alterations. The colorful alterations were observed to originate from corrosion products of bronzeware. The green, black, yellow, blue, purple, and white alterations were due to malachite, tenorite, pyromorphite, azurite, diaboleite, and cassiterite, respectively. Meanwhile, organic matter and hypertoxic arsenolite were first found on ancient jade artifacts.

## Introduction

Ancient jade artifacts, which are among the most characteristic relics of the ancient Chinese civilization, constitute a significant cultural symbol of China^1^. Jade artifacts served as decorative items in the ancient Chinese society, and hold rich cultural value in the Chinese culture, as they represent status and power. Jade artifacts have been associated with the ancient civilizations of more than 20 countries or regions throughout the world, including those of the Maoris in New Zealand, the native Americans in North America, and the Mayas in South and Central America^2,3^. However, the use of jade ware in the Chinese culture is the only one that has lasted more than 8000 years without interruption.

In recent decades, research on jade items has become an active field since many impressive ancient jade artifacts have been unearthed throughout China. It has extremely improved China’s status in academia as well as its social influence in the domain of archaeology. Studies of ancient Chinese jade artifacts include investigation of the jade materials and their alterations, forms, styles, carving techniques, functions, and cultural significance associated with such relics.

Research on alterations found on jade artifacts has received the recent interest attention of archaeologists and other scholars who would like to understand the burial history of these objects. “Alteration” geologically refers to chemical composition and/or structural changes of minerals under the influences of hydrothermal fluids, surface water, seawater, or other environmental conditions. In this paper, we use the word “alteration” to refer to chemical component and structural changes in jade artifacts caused by human activity and natural weathering, which is different from the term in geology. It is an important and typical characteristic of ancient jade artifacts recovered through archaeological excavations^4–6^. Furthermore, such jade alterations encompass abundant archaeological information. Alteration of ancient jade artifacts is manifested in seven colors, each of which has multiple mechanistic origins^1^. Some scholars such as Casadio et al.^7^ and Chen et al.^8^ have previously studied alterations of ancient jade artifacts using Raman spectroscopy. Macroscopic observations have shown that almost all of ancient jade artifacts have undergone alteration. It is known that the deposited corrosion products of bronzeware signify an important type of alteration of ancient jade artifacts. However, few in-depth studies have been undertaken to investigate the alteration caused by the corrosion products of bronze, owing to which archaeological research and conservation efforts have been limited. In this study, we used nondestructive analysis to examine the color alteration produced due to bronze corrosion of ancient jade artifacts and discuss further problems to focus upon.

## Experimental

### Artifacts

**Figure 1:**
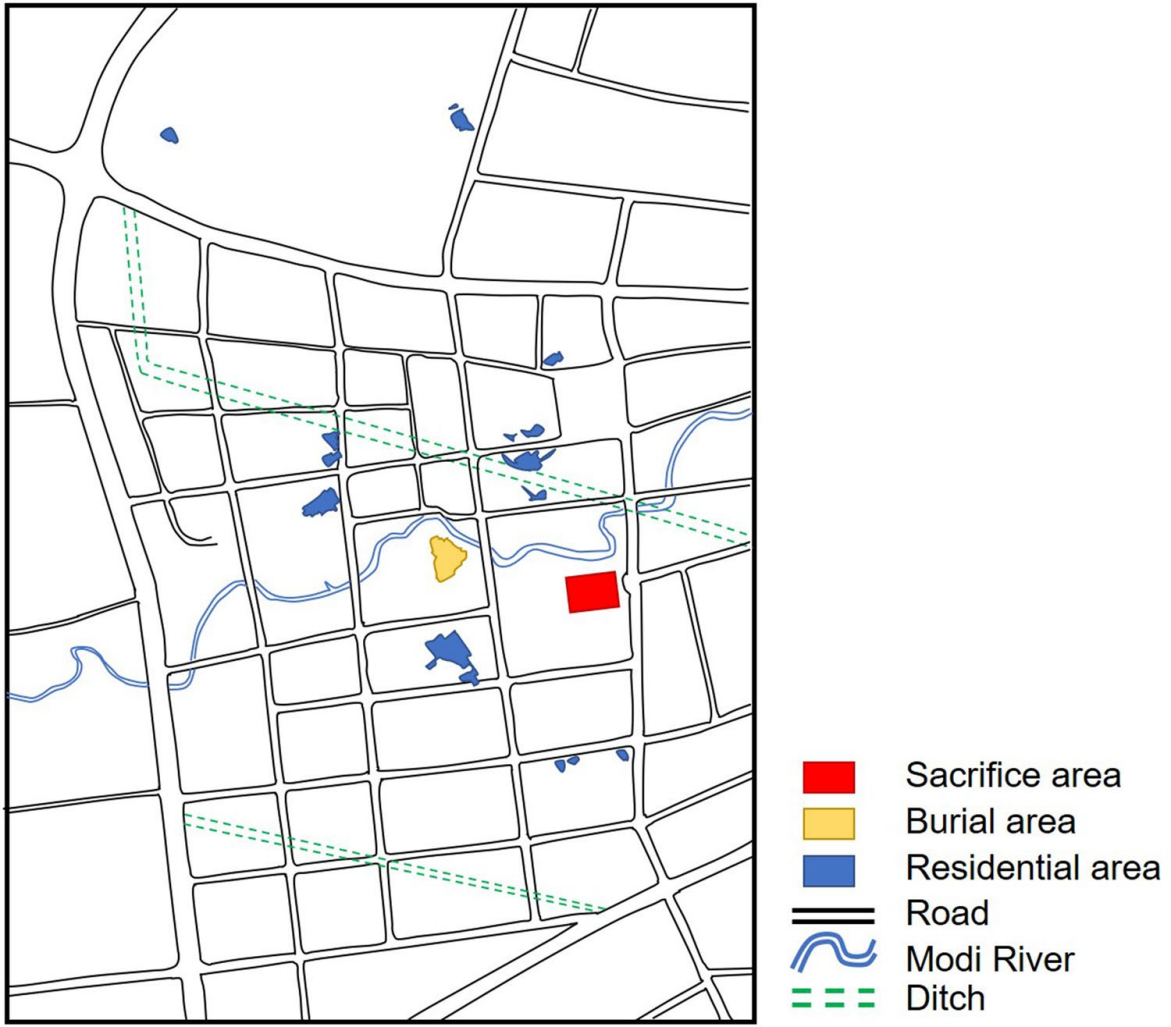
Plane graph of archaeological sites in the Jinsha Site. The sacrifice area is the Meiyuan sacrificial area.

The ancient jade artifacts studied in this work were unearthed from the Jinsha Site. The Jinsha Site at Chengdu, Sichuan Province, dating back from the Shang Dynasty to the Spring and Autumn period (3300–2600 years BP), was excavated in 2001^9^. The discovery of the Jinsha site has revived the lost glory of the ancient Shu kingdom and Shu culture and has provided convincing explanation for the abrupt disappearance of the Sanxingdui culture^10^. The Jinsha Site can be classified into three kinds of regions according to their function: the sacrifice area, burial area, and residential area (Fig. 1). A sacrifice area referred to as the Meiyuan Site is well-known for its numerous unearthed jade, bronze, and ivory artifacts. The ancient jade artifacts studied in this work were unearthed from the Meiyuan site^11^.

**Figure 2:**
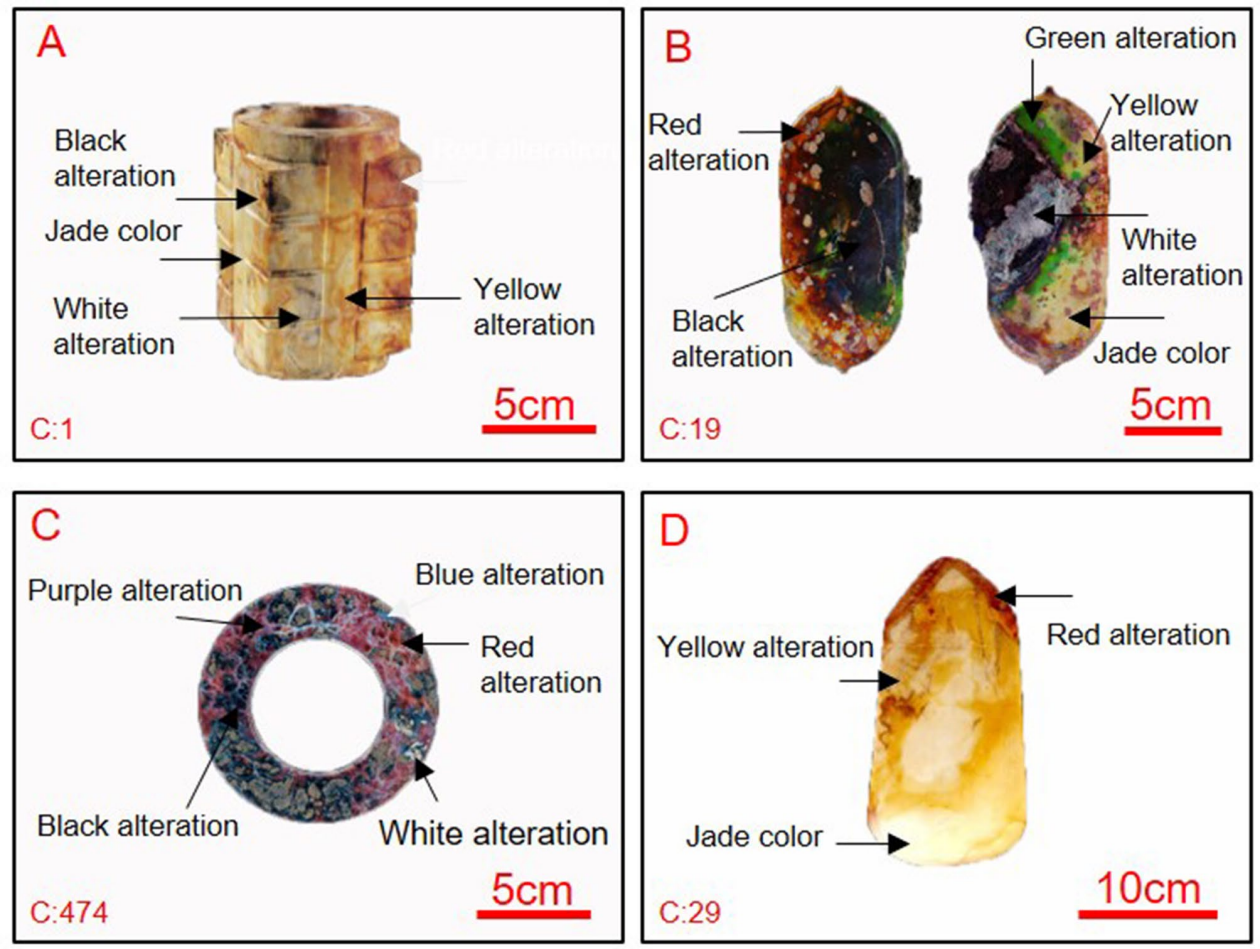
Photographs of ancient jade artifacts unearthed from the Meiyuan sacrificial area of the Jinsha Site. (**A**) Four-node cong; (**B**) oval-shaped jade artifact; (**C**) collared bi disk; (**D**) battle-axe with the pattern of an animal’s face.

Representative and notable colors of jade artifacts unearthed from the Meiyuan site are shown in Fig. 2. The impressive colors found on ancient jade artifacts, which cover all color variations related to jade alteration including yellow, red, green, white, black, blue, or purple, are attributable to their alteration^12^. The four-node cong in Fig. 2A shows yellow, red, black, and white alterations. The oval-shaped jade artifact (Fig. 2B) exhibits green, red, yellow, black, and white alterations. Collared bi disks (Fig. 2C) show blue, red, white, and black alterations. The battle-axe with animal-face pattern (Fig. 2D) has red and yellow alterations. In this study, we focused on six color alterations of ancient jade artifacts unearthed from the Meiyuan Site of the Jinsha Site. Alterations of fourteen pieces of ancient jade artifacts fragments have been studied in this paper as listed in Table 1.

**Table 1:** Material type and alteration type of the ancient jade artifacts.

	Artifact Number	Material type	Alteration type
1	JS-1	Nephrite	Blue, white
2	JS-2	Nephrite	Blue
3	JS-3	Nephrite	Black, yellowish-brown
4	JS-4	Nephrite	White, black, purple, brownish-red, yellowish-brown
5	JS-5	Nephrite	White, black, purple, brownish-red, yellowish-brown
6	JS-6	Nephrite	White, black, gray, dark purple
7	JS-7	Nephrite	Gray, white, brownish-red
8	JS-8	Nephrite	Brown, white
9	JS-10	Nephrite	White, black, purple, brownish-red, yellowish-brown
10	JS-11	Nephrite	Reddish-brown, white, yellowish-brown
11	JS-12	Nephrite	White
12	JS-13	Nephrite	White
13	JS-14	Nephrite	White
14	JS-15	Nephrite	White

Jade JS-9 was not included in this study.

### Instrumentation and methods

Considering the rarity and irreducibility of cultural relics, in this work, we used the nondestructive methods of Raman spectroscopy, optical microscopy (OM), and scanning electron microscopy coupled with energy-dispersive spectroscopy (SEM–EDS). Raman spectroscopy facilitates mineral phase analysis, whereas OM and SEM enable the analysis of microstructures. EDS is used to determine the chemical composition of the substrate.

Raman spectroscopic analysis was undertaken at the State Key Laboratory of Polymer Molecular Engineering at the Fudan University. The instrument (XploRA) used was a laser micro-Raman spectrometer produced by Horiba Scientific Company (Japan). The type of laser source used in the measurement were 633 nm, 17 mW, helium–neon laser; and 785 nm, 30 mW, semiconductor laser. All spectra were obtained at room temperature at the spectral resolution of 1 cm^−1^, and a 20× objective lens was used often.

SEM–EDS was carried out on a Phenom XL G2 (Phenom Scientific, the Netherlands), which is a desktop scanning electron microscope with ~ 8 nm resolution. The machine was used for structural analyses and chemical composition. The test proceeded under 15 kV and 10 Pa.

## Results and discussion

### Investigation of constituent material of ancient jade artifacts

Phase identification of the ancient jade artifacts of the Jinsha Site is necessary because their appearances are not like those of normal jade artifacts that are usually white or green in color. Raman spectroscopy was employed to explore the mineral phases of these artifacts.

**Figure 3:**
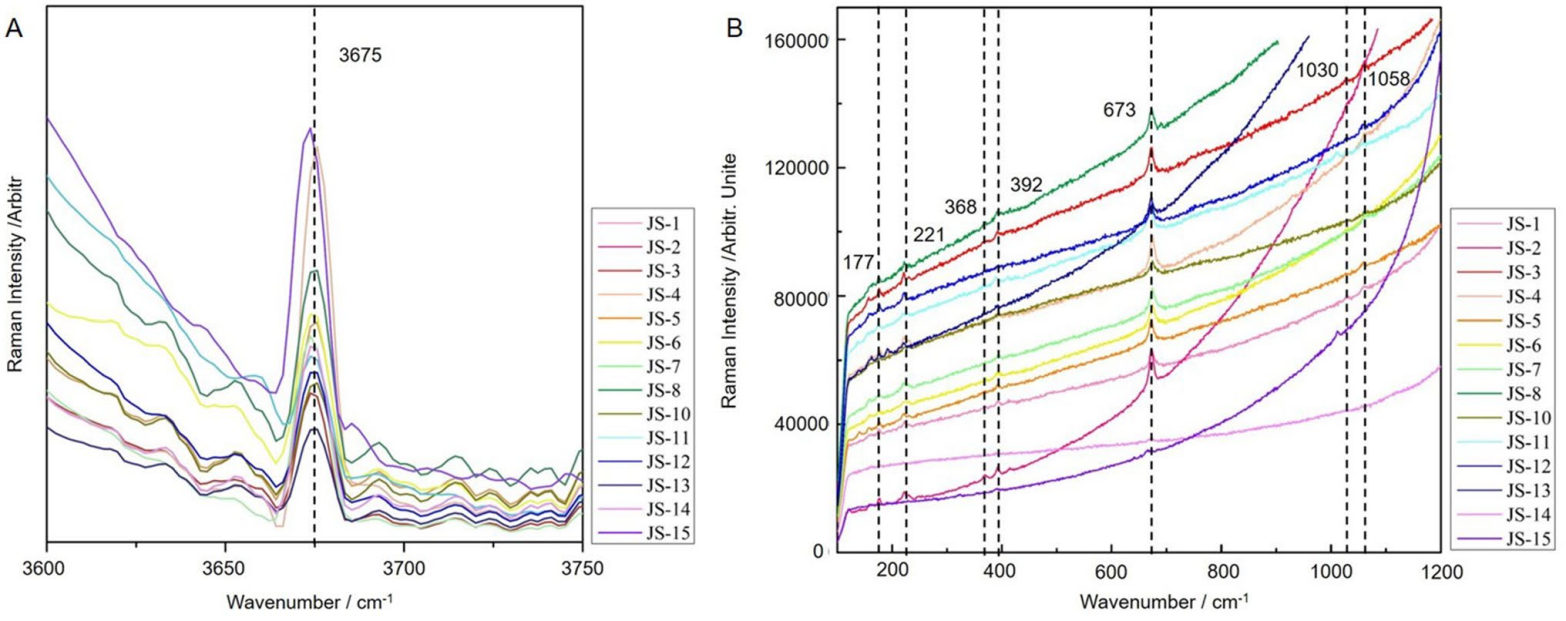
Raman spectra acquired from ancient jade artifacts unearthed from the Meiyuan sacrificial area of the Jinsha Site. (**A**) In the range of 100–1200 cm^−1^. The material of each is identified as tremolite nephrite. (**B**) In the range of 3600–3750 cm^−1^.

Figure 3A shows the Raman spectra acquired from the jade artifacts in the range of 100–1200 cm^−1^. These artifacts exhibit similar spectra. The data show vibrations of the silicon–oxygen bonds, metal–oxygen bonds, and the lattice. Specifically, the peak at 674 cm^−1^ is the characteristic peak of nephrite jade [Ca_2_(Mg, Fe)_5_Si_8_O_22_(OH)_2_], corresponding to the symmetric stretching vibrations of Si–O_b_–Si bridges. During on-site testing, the 674 cm^−1^ peak is often the only one observed in the Raman spectra of many ancient nephrite jade artifacts. The bands observed at 1030 and 1059 cm^−1^ can be attributed to the antisymmetric stretching vibrations of Si–O_b_–Si bridges. The band at 221 cm^−1^ was attributed to the vibrations of O–H–O groups. The assignment of the observed bands was problematic due to the coupling of the vibrations of the MO_6_ and MO_8_ polyhedra (M: cations in octahedral or cubic sites), the deformation modes of the (Si_4_O_11_) ribbons, and the F–O bonds^13^.

Figure 3B shows the Raman spectra of ancient jade artifacts in the range of 3600–3750 cm^−1^. According to a former study^14^, a Raman spectrum could be used to distinguish tremolite from actinolite, both of which belong to the same series of amphibole minerals, according to the number and location of vibrational peaks in the 3600–3750 cm^−1^ range. Tremolite yields one Raman peak, while actinolite yields two or three peaks in this region. The main minerals present on the artifacts were all determined to be tremolite as they only exhibited one peak in the 3600–3750 cm^−1^ region.

Nephrite [Ca_2_(Mg, Fe)_5_Si_8_O_22_(OH)_2_], a fine-grained, compact variety of tremolite, is the main jade material studied in this work. Moreover, many kinds of materials of jade artifacts are found in the Jinsha Site, including tremolite, actinolite, diopside, plagioclase, serpentine, talc, marble, pyrophyllite, turquoise, and agate. However, tremolite is the most important jade material^12^.

### Green alteration

Green alteration is the most famousalteration noted on ancient jade artifacts unearthed from the Jinsha Site. However, it is the most undistinguishable alteration because it resembles the original green color of the jade material.

**Figure 4:**
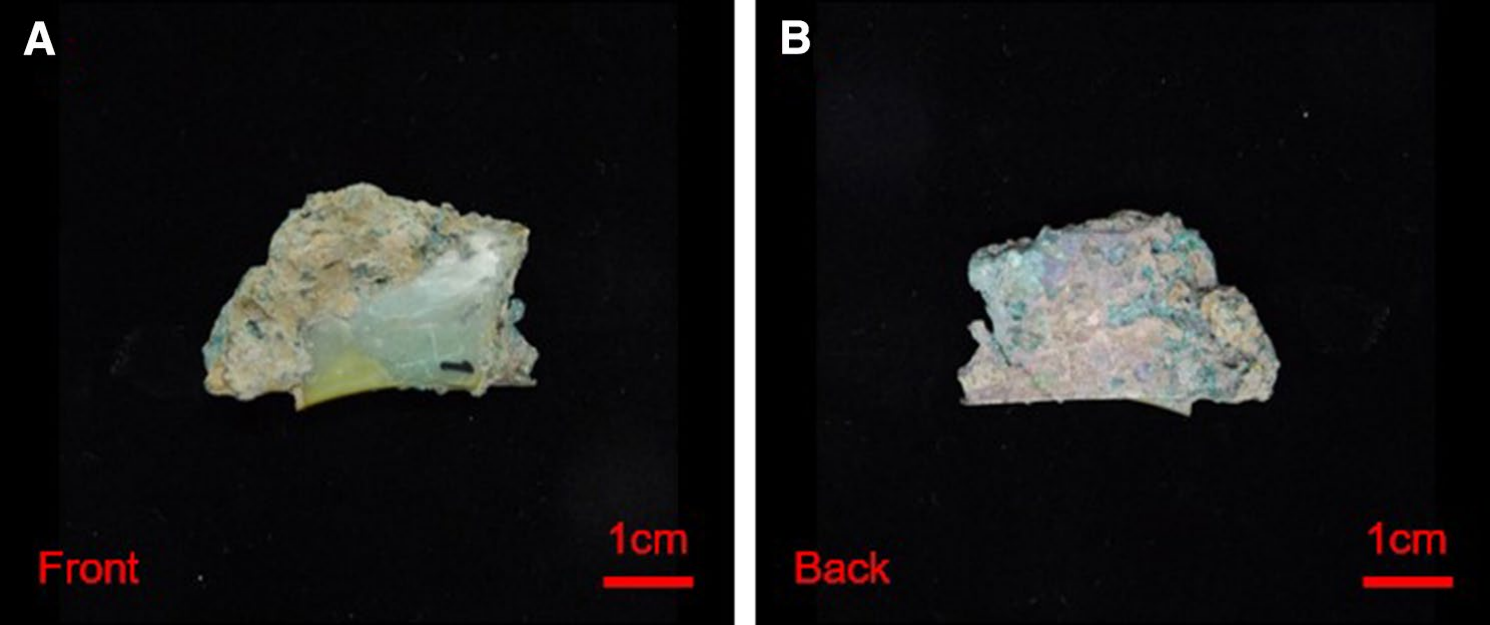
Photo of ancient jade artifact JS-1.

JS-1, an ancient jade artifact with green alternation, was unearthed from the Jinsha Site (Table 2). This artifact has two parts: a piece of jade (Fig. 4A) and a piece of bronze (Fig. 4B). The jade part and bronze part are connected by earth. This connection protected the space relationship between the jade and bronze components over the period of burial. First, the jade part shows various colors: the main color is green with a little blueish hue, and there were some areas with small pieces of yellowish-green color were observed on the jade surface. However, the distribution of the natural green color of jade material rarely shows this feature. These two kinds of green colors may belong to the jade material and green coloration due to alteration, respectively. It is difficult to distinguish the jade material from the alteration without Raman spectral analysis since these two kinds of green colors have been found on natural nephrite jade materials. Second, the bronze part had a rough surface with earthy luster and opacity. Some regions were green, black, reddish-brown, grey, white, and yellowish-brown in color.

**Table 2:** Physical characteristics of ancient jade fragment JS-1.

Artifact	Basic characteristics			Alteration characteristics			Jade characteristics	
	Color	Glossiness	Transparency	Color	Distribution	Degree	Color	Structure
JS-1	Green is the main color; shows some yellowish-green and white coloring	Greasy luster	Subtranslucent	Green, white	Green in face shape and white in dot shape	Medium	Green	Compact

**Figure 5:**
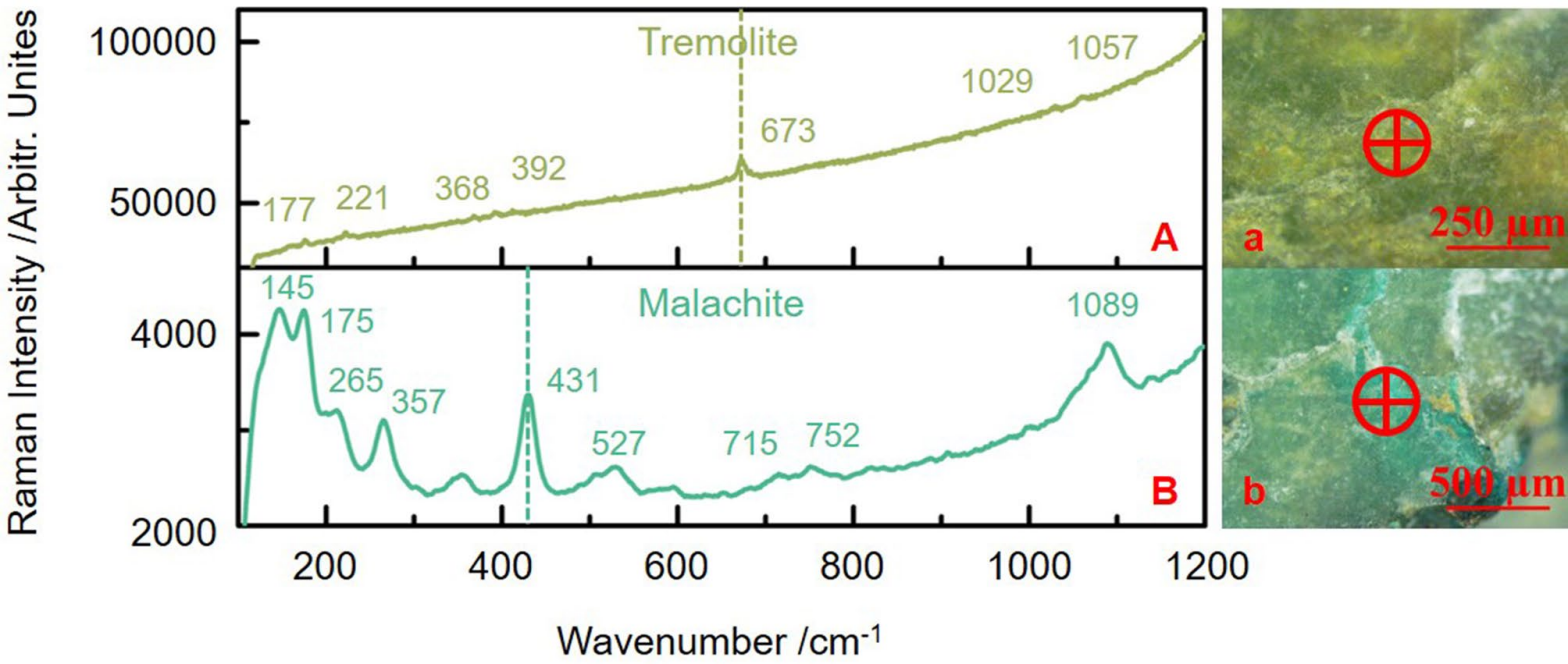
Raman spectra of green alteration and jade material of JS-1. (**A**) tremolite jade material, (**B**) malachite alteration. Micro-photos and test points: (a) tremolite jade material, (b) malachite alteration.

Figure 5 shows the Raman spectra of jade material (Fig. 5A, yellowish-green part) and green alteration (Fig. 5B, bluish-green part) of JS-1. Raman spectrum of the yellowish-green part showed typical tremolite characteristics with the characteristic 673 cm^−1^ peak. Raman spectrum of the bluish-green part showed typical peaks of malachite [Cu_2_(CO_3_)(OH)_2_]. The characteristic intense band at 430 cm^−1^ was attributed to the Cu–O group in malachite. In the high-wavenumber range (above 700 cm^−1^), the 1089 cm^−1^ peak is attributed to the symmetric stretching mode of the carbonate group in malachite. In the low-wavenumber range (below 600 cm^−1^), three peaks due to the lattice modes of malachite are observed at 175, 265, and 357 cm^−1^^15^.

Malachite is one of the main green corrosion products of bronzeware. Malachite on nephrite jade arises from the corrosion of bronzeware. Malachite was also found on the bronze part of JS-1, reconfirming the source of malachite on the ancient jade artifacts. The green malachite was found to be the primary source of green alteration observed on the ancient jade artifacts unearthed from the Jinsha Site.

First, malachite alteration could cover almost the entire surface area of the ancient jade artifacts. The malachite alteration contributes to the main color of JS-1 and encompasses almost 90% of the surface, as shown in Fig. 4A. Meanwhile, the yellowish-green color of the nephrite jade material of JS-1 only covered about 10% of the surface. Considering this proportion, it is challenging to distinguish the jade material and malachite alteration only according to the occupied area. Furthermore, it is hard to judge whether the jade has alteration when malachite alteration covers the entire surface of jade artifacts.

**Figure 6:**
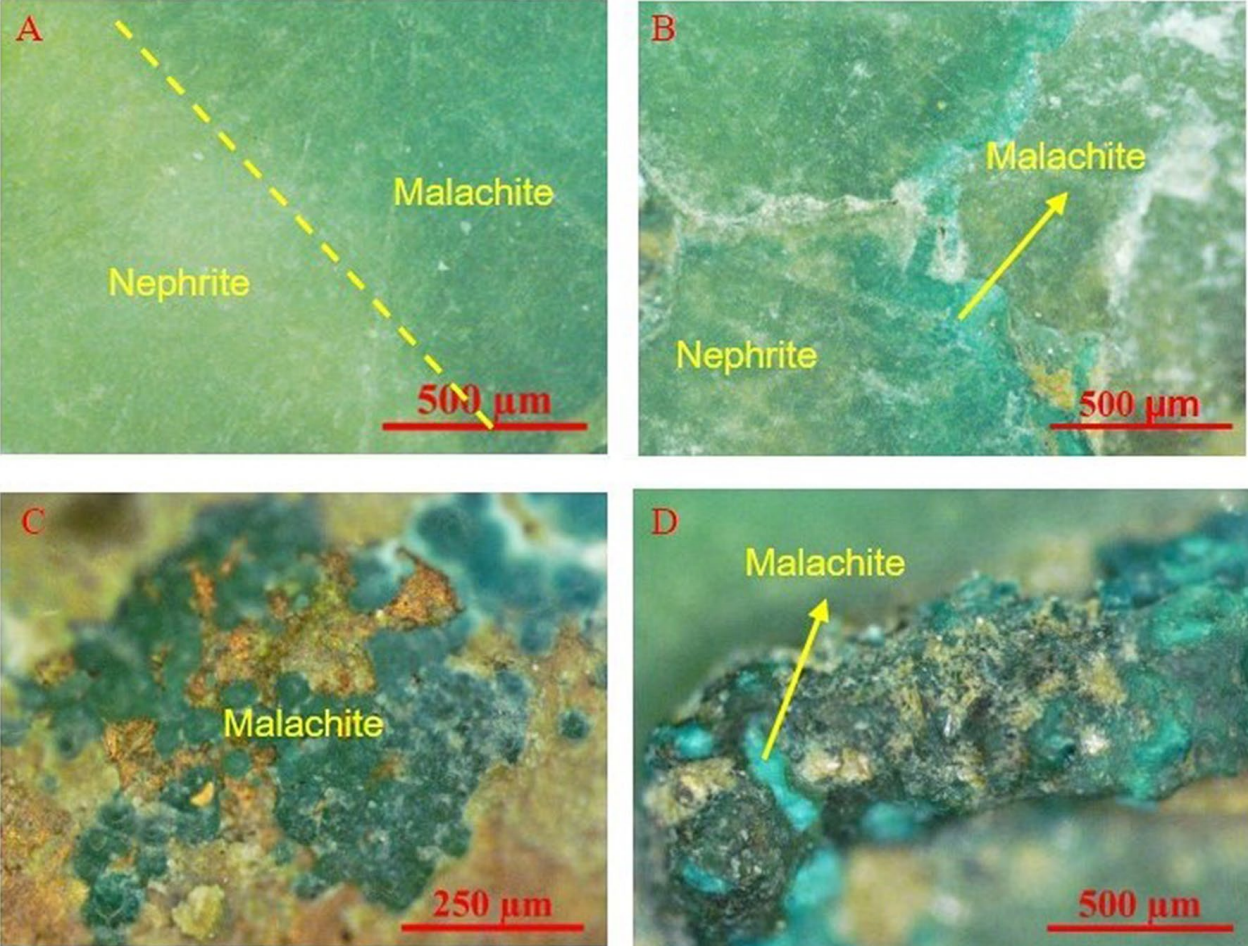
Optical micrographs of JS-1 ancient jade artifact with green alteration.

Secondly, the distribution of malachite alteration on ancient jade artifacts showed a particular kind of face shape, as shown in Fig. 6A. The bluish-green color of malachite alteration naturally transitioned with the yellowish-green color of the jade material. The thickness of malachite alteration in the micrometer range contributed to the high degree of difficulty in interpretation as well. The distribution of malachite in the face shape was so natural that it was hard to distinguish from the jade material through OM observation only (Fig. 6A).

Thirdly, the malachite alteration showed different hues of green, as shown in Fig. 6B–D. The color of malachite, such as different hues of bluish-green, yellowish-green and green, is related to the content of different kinds of coloring elements in the mineral^16^. This augments the difficulty in distinguishing the alteration when the green color of malachite alteration is similar to that of the original jade material. At the same time, the various hues of the green color of malachite alteration make the alteration more complicated.

Fourthly, the different hues of bluish-green, yellowish-green, and green colors are natural colors observed for the nephrite jade material^17,18^. The appearance of the bluish-green, yellowish-green, or green color on ancient jade artifacts does not indicate the presence of malachite alteration. Ancient jade artifacts that have malachite alteration of a similar green color constitute the toughest objects of study in the investigation of the alteration of ancient jade artifacts.

### Other five colors of alterations

**Figure 7:**
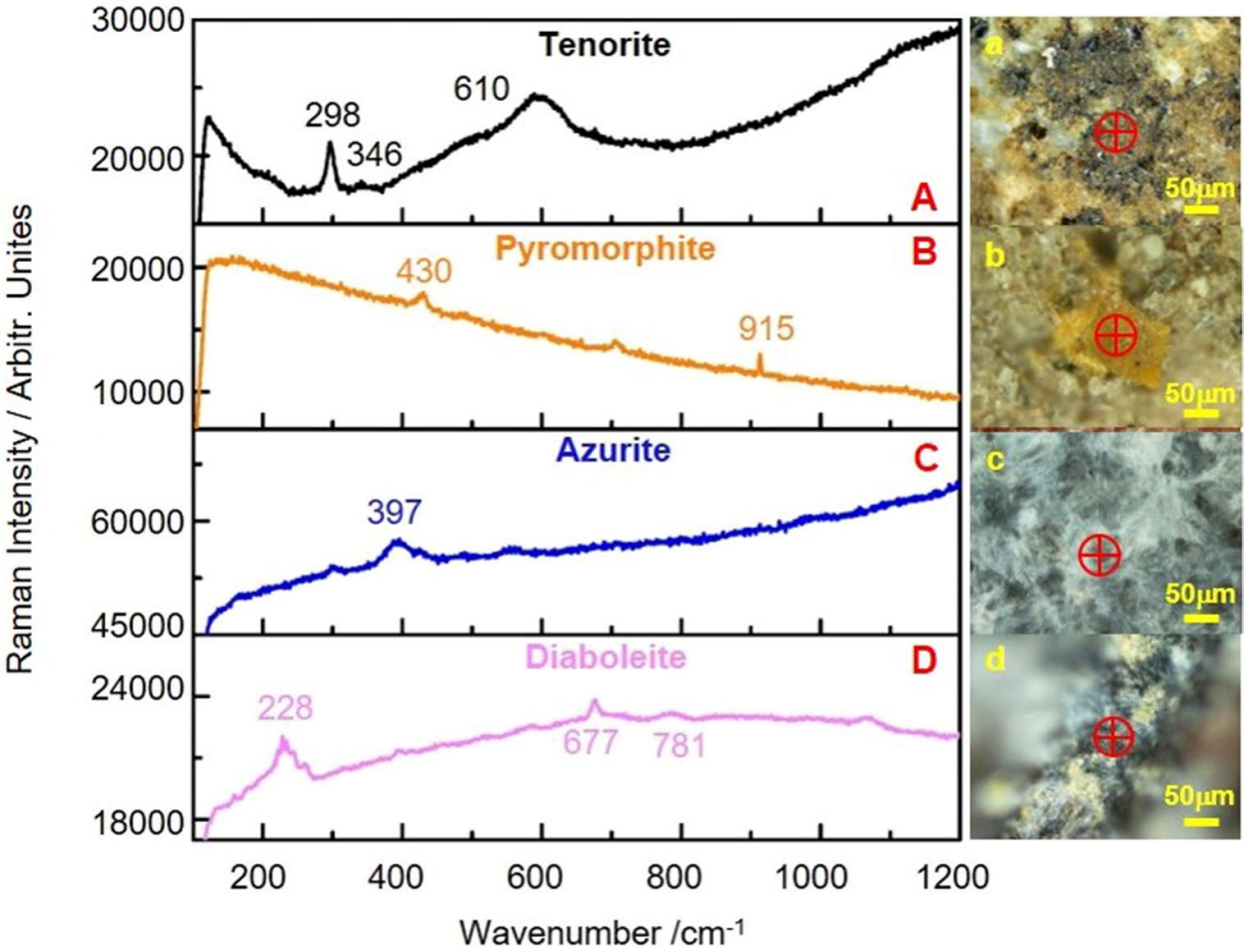
Raman spectra for regions showing alterations of 4 colors on ancient jade artifacts: (**A**) black alteration, (**B**) yellow alteration, (**C**) blue alteration, and (**D**) purple alteration. The corresponding optical micrographs are shown in the right panel. The test point is marked with an encircled cross in each micrograph.

The other five colors of alterations noted on ancient jade artifacts unearthed from the Jinsha Site are black, yellow, blue, purple, and white. Four of these were investigated by Raman spectroscopy. First, black alteration was due to one kind of tenorite mineral (CuO). Tenorite is the primary contributing mineral leading to black alterations. The Raman band at 298 cm^−1^ is the characteristic band of tenorite (Fig. 7A). Second, the yellow alteration mainly pyromorphite (Pb_5_(PO_4_)_3_Cl). The Raman spectrum of pyromorphite is shown in Fig. 7B. The Raman bands at 430 and 915 cm^−1^ are the characteristic bands of pyromorphite. Third, the blue alteration is attributable to one kind of mineral, azurite (Cu_3_(CO_3_)_2_(OH)_2_). The Raman spectrum of azurite shown in Fig. 7C shows an intense band at 397 cm^−1^, attributable to the Cu–O group of azurite^15^. Azurite is always found in the oxidized zones of copper. Fourth, the purple alteration is due to diaboleite (Pb_2_CuCl_2_(OH)_4_), which is a secondary mineral found in deeply oxidized Pb–Cu ores. The Raman spectrum of diaboleite is shown in Fig. 7D. The band observed at 227 cm^−1^ is the most intense band that has previously been assigned to OH–O hydrogen bonding vibrations. Two hydroxyl deformation mode vibrations appeared at 781 and 677 cm^−1^^19^.

**Figure 8:**
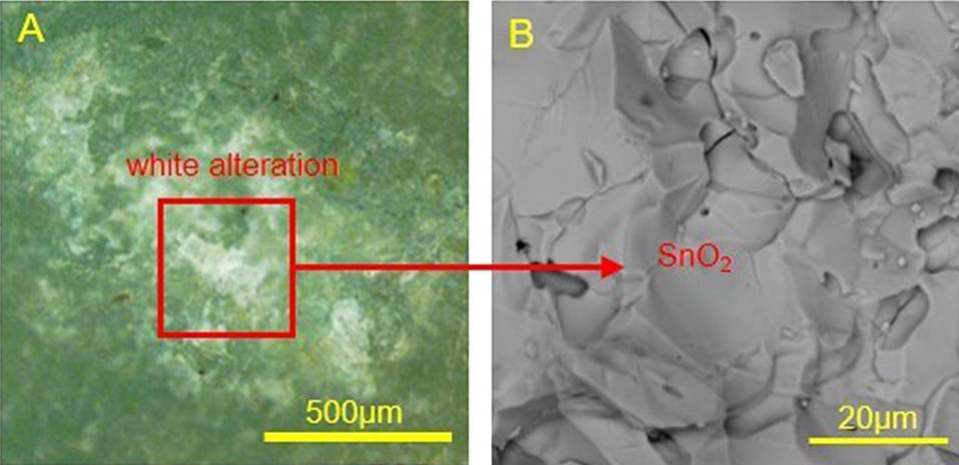
Optical micrograph (**A**) and SEM image (**B**) of the white alteration on JS-1.

The white alteration material showed no Raman signal. SEM–EDS data revealed that the white alteration was mainly due to a material comprising Sn (77.6%) and O (22.4%). SEM–EDS images depicting the micro-morphology of the white alteration are shown in Fig. 8. The white alteration was determined to be cassiterite (SnO_2_) by calculations.

Seven kinds of alterations originate from copper materials. They are common copper corrosion products that always stay on the surface or inside the micro-pores of jade material. However, corrosion products also contain many other kinds of mineral phases^19^. Ancient jade artifacts were buried with numerous copper artifacts according to the archaeological excavation report produced for the Jinsha Site^9^. It is reasonable to assume that the alterations in ancient jade artifacts are related to these copper artifacts.

### Other materials in ancient jade artifacts

**Figure 9:**
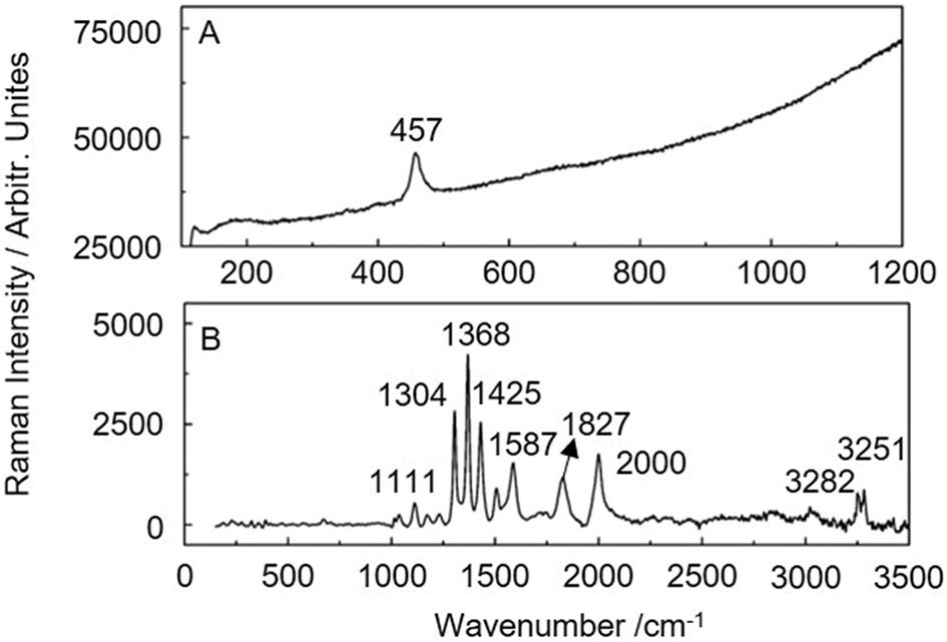
Raman spectra of other materials on ancient jade artifacts: (**A**) arsenolite and (**B**) organic matter.

Raman spectroscopy could be used in further studies of ancient Chinese jade artifacts. Figure 9A shows that arsenolite (As_2_O_3_) was present on ancient jade artifacts unearthed from the Jinsha Site. As is an element commonly found in bronzeware and has also been detected in bronzeware unearthed from the Jinsha Site. Arsenolite is a corrosion product of bronzeware and could be considered as an alteration of ancient jade artifacts. However, arsenolite is hypertoxic. Researchers should therefore pay attention to safety measures such as wearing gloves and protecting themselves adequately when arsenolite is assumed to be possibly present on ancient jade artifacts. Ancient jade artifacts with alterations originating from corrosion products of bronzeware should be carefully examined to ensure the safety of researchers.

During the alteration study, some organic matter was also found on ancient jade artifacts unearthed from the Jinsha Site. The source of organic matter may be the materials present in their surrounding environment, such as organic matter in soil or organic matter buried with the artifacts. Several ancient jade artifacts studied in this work yield the Raman spectra in Fig. 9B. The Raman spectra show a peak at 1111 cm^−1^, indicating N–N stretching vibrations. The in-plane deformation of CH_2_ results in strong peaks at 1304 and 1368 cm^−1^. The peaks at 1425 and 1587 cm^−1^ are attributed to the vibrations of C=C. The one at 1827 cm^−1^ is assigned to the stretching vibrations of C=O. The 2000 cm^−1^ peak is the Raman peak of the C=C groups. The peaks at 3282 and 3251 cm^−1^ indicate the vibrations of NH_2_ or $\equiv \mathrm{CH}$. This is the first time that organic matter has been found on ancient jade artifacts. This phenomenon reminds ancient jade researchers to be more careful when studying unearthed ancient jade artifacts. And we should choose lower energy test parameters to protect ancient jade artifacts when doing scientific tests.

Table 3 lists the band positions for various alterations detected on the ancient jade artifacts unearthed from the Jinsha Site in this study.

**Table 3:** Peak positions in the Raman spectra of different mineral phases of alteration of ancient Chinese jade artifacts.

	Mineral phases	Chemical formula	Major Raman band positions (cm^−1^)	Ancient jade artifact
1	Tremolite	Ca_2_(Mg, Fe)_5_Si_8_O_22_(OH)_2_	177, 221, 368, 392, 673, 1029, 1057	All artifacts
2	Malachite	Cu_2_(CO_3_)(OH)_2_	145, 175, 265, 352, 431, 527, 715, 752, 1089	JS-2
3	Tenorite	CuO	298, 346, 610	JS-1
4	Pyromorphite	Pb_5_(PO_4_)_3_Cl	430, 915	JS-13, JS-15
5	Azurite	Cu_3_(CO_3_)_2_(OH)_2_	397	JS-7
6	Diaboleite	Pb_2_CuCl_2_(OH)_4_	228, 677, 781, 1070	JS-6
7	Arsenolite	As_2_O_3_	457	JS-2

The general methods used to study the alterations of ancient jade artifacts are not suitable for recognizing and distinguishing alterations from the jade materials. Thus, Raman spectroscopy is the best method to study the alterations of ancient jade artifacts. Meanwhile, it plays an irreplaceable role in the study of alterations of jade artifacts because it is not only a rapid, accurate, and nondestructive technique but also low requirement for artifacts. At the same time, it can also provide additional information on other materials (e.g., organic matter) present on such ancient jade artifacts.

## Conclusions

This work uses nondestructive analysis investigate the alteration observed on ancient jade artifacts unearthed from the Jinsha Site in China. Firstly, this research identified that the jade material of ancient jade artifacts is nephrite and the main mineral is tremolite. Secondly, this work investigated six color alterations: green, black, yellow, white, blue, and purple. The constituent materials were found to be malachite, tenorite, pyromorphite, cassiterite, azurite, and diaboleite, respectively. Thirdly, the nondestructive technique helped determine the presence of the hypertoxic material, arsenolite, which can be of relevance in future research in the preservation of cultural relics and protection measures for researchers. Fourthly, organic matter was found on the surface of ancient jade artifacts for the first time in this work.

In archeological studies, nondestructive analysis is an important principle. Raman spectroscopy is one of the best methods for analyzing the phase structure of ancient artifacts because it is the best technique for noncontact original position analysis without artifact preparation, especially in the cases of alterations of ancient Chinese jade artifacts. This is the first work to use Raman spectroscopy to study such colorful alterations, which comprise a characteristic feature of ancient Chinese jade artifacts. Raman spectroscopy can provide plentiful information. First, Raman spectroscopy could be used to identify the jade material rapidly and accurately without artifact preparation. Second, it could be used to investigate different kinds and origins of alterations. Third, the Raman technique combined with SEM–EDS revealed that the alteration materials to exhibit poor crystallinity and thus the need for better conservation of the jade artifacts. Lower energy and lower destructive test conditions is the best choice in the process of scientific research. Fourth, Raman analysis facilitated the detection of small quantities of organic materials and hazardous substances that had not been detected prior to this. Thus, we believe the Raman technique is advantageous in to study alterations on ancient Chinese jade artefacts and can be advantageously used in the examination of various other materials in art, archaeology, and culture heritage materials.
